# Technology-Based Program Effects on Health Outcomes and Caregiving Experience in Asian American Caregivers

**DOI:** 10.1093/geroni/igaf122.3853

**Published:** 2025-12-31

**Authors:** Jiwon Baek, Dongmi Kim, Seulgi Ryu, Yu-Shan Cheng, Wonshik Chee, Eun-Ok Im

**Affiliations:** University of Texas at Austin, Austin, Texas, United States; University of Texas at Austin, Austin, Texas, United States; The University of Texas at Austin, Austin, Texas, United States; University of Texas at Austin, Austin, Texas, United States; University of Texas at Austin, Austin, Texas, United States; University of Texas at Austin, Austin, Texas, United States

## Abstract

**Background:**

Midlife women caring for people living with Alzheimer disease face substantial burdens that jeopardize health. Such burdens are often greater among ethnic minority women due to cultural norms surrounding illness and caregiving. We hypothesized that a culturally tailored and technology-based program would improve (i) health outcomes of caregivers and care recipients and (ii) caregiving experience.

**Methods:**

This study developed a 3-month intervention program based on Bandura’s Self-Efficacy Theory of Behavior Change and delivered a coaching/support intervention program via a project platform and the Alzheimer’s Association website. Although the study used random assignment, analyses were restricted to the intervention group due to an underpowered control group. This preliminary analysis included 30 caregivers with repeated measures of caregivers’ and care recipients’ health outcomes (stress and physical and psychological symptoms of caregivers; cognitive, mood, and quality-of-life status of care recipients) and caregiving experiences (caregiving activity, caregiving competence, and coping and behavioral skills). Linear mixed-effects panel models were estimated with adjusting for sociodemographic and health/disease-related covariates.

**Results:**

Caregivers demonstrated statistically significant improvements following the technology-based interventions. Perceived stress and acculturation stress of caregivers decreased. Although behavioral and psychological symptoms of care recipients showed no significant change, caregivers’ reaction to their symptoms decreased. Both care recipients’ mood and caregivers’ caregiving skills showed significant improvement.

**Conclusions:**

A 3-month information and coaching/support intervention was associated with meaningful short-term improvements in caregiver’s and care recipients’ health and caregiving experience. Findings support the potential of culturally aligned interventions to reduce health disparities among ethnic minority women caregivers.

